# Varanoid Tooth Eruption and Implantation Modes in a Late Cretaceous Mosasaur

**DOI:** 10.3389/fphys.2016.00145

**Published:** 2016-05-17

**Authors:** Min Liu, David A. Reed, Giancarlo M. Cecchini, Xuanyu Lu, Karan Ganjawalla, Carol S. Gonzales, Richard Monahan, Xianghong Luan, Thomas G. H. Diekwisch

**Affiliations:** ^1^Department of Periodontology, Stomatological Hospital, Jilin UniversityChangchun, China; ^2^Department of Oral Biology, University of Illinois, Chicago, IL, USA; ^3^College of Dentistry, Midwestern University, Downers Grove, IL, USA; ^4^School of Dental Medicine, Harvard University, Boston, MA, USA; ^5^Department of Oral Diagnosis, University of Illinois College of Dentistry, Chicago, IL, USA; ^6^Center for Craniofacial Research and Diagnosis and Department of Periodontics, Texas A&M University Baylor College of DentistryDallas, TX, USA

**Keywords:** tooth eruption, tooth replacement, resorption pits, reptilian phylogeny, mosasaurs

## Abstract

Erupting teeth are some of the oldest witnesses of developmental processes in the vertebrate fossil record and provide an important resource for vertebrate cladistics. Here, we have examined a mosasaur jaw fragment from central Texas using ultrathin ground section histology and 3D tomographic imaging to assess features critical for the cladistic placement of mosasaurs among varanoids vs. snakes: (i) the orientation of replacement teeth compared to the major tooth axis, (ii) the occurrence of resorption pits, and (iii) the mode of tooth implantation/attachment to the tooth bearing element (TBE). The replacement tooth studied here developed in an inclined position slightly distal of the deciduous parent tooth, similar to another varanoid squamate, the Gila monster *Heloderma suspectum*. Ground sections and tomographs also demonstrated that the replacement tooth attachment apparatus was entirely intact and that there was no evidence of mechanical deformation. Sections and tomographs further illustrated that the replacement tooth was located within a bony crypt and the inclination of the crypt matched the inclination of the replacement tooth. These preparations also revealed the presence of a resorption pit within the boundaries of the deciduous tooth that surrounded the developing replacement tooth. This finding suggests that developing mosasaur teeth developed within the walls of resorption pits similar to varanoid tooth germs and unlike developing snake teeth which are surrounded by fibrous connective tissue integuments. Finally, mosasaurs featured pseudo-thecodont tooth implantation with teeth anchored within a socket of mineralized tissue by means of a mineralized periodontal ligament. Together, these data indicate that the moderate inclination of the erupting mosasaur tooth studied here is neither a result of postmortem displacement nor a character representative of snakes, but rather a shared character between Mosasaurs and other varanoids such as *Heloderma*. In conjunction with the presence of resorption pits and the evidence for pseudothecodont tooth implantation, the tooth eruption and implantation characters described in the present study either place mosasaurs among the varanoids or suggest convergent evolution mechanisms between both clades, with mosasaurs evolving somewhat independently from a common varanoid ancestor.

## Introduction

Mosasaurs are an extinct group of large marine squamates that dominated mid- and late- Cretaceous oceans some 65–100 million years ago (MYA, *Clidastes*, Luan et al., 2009a). The phylogenetic classification of these large marine predators has been the subject of an animated scientific debate for centuries. The majority of scholars consider mosasaurs to belong to an evolved superfamily of squamates, the Varanoidea, which includes besides the Mosasauridae also a number of extant lizards such as the Komodo dragon (*Varanus komodoensis*) and the Gila monsters (*Heloderma*; Carroll, 1988). Most recent integrated analyses place mosasaurs within Toxicofera as a sister group of snakes (Reeder et al., 2015). Mosasaur-snake affinities go back to earlier theories based on a number of properties of the mosasaur dentition, including tooth attachment and tooth replacement (Cope, 1869, 1872; Owen, 1877, 1878; Caldwell and Lee, 1997; Lee, 1997a,b; Zaher and Rieppel, 1999; Tchernov et al., 2000; reviewed in Greene and Cundall, 2000; Rieppel et al., 2003), re-awakening a century-old concept of an aquatic origin of snakes first introduced by Cope (1872). Cope noted certain similarities in the feeding apparatus between mosasaurs and macrostomatan snakes, including a separation between the mandibular rami allowing for feeding on large prey (Luan et al., 2009b). Lee (1997a,b) returned to Cope's original hypothesis, largely based on similarities in tooth eruption patterns between mosasaurs and snakes, suggesting that snakes and mosasaurs are derived from a common ancestor characterized by recumbent replacement teeth, which he thought of as the most important character (Lee, 1997a). He believed that these characters were in favor of an aquatic origin of snakes, when compared to the classic terrestrial burrowing theory (Lee, 1997b; Caldwell and Lee, 1997). However, most recent fossil discoveries and analyses do not support a marine origin of snakes based on the placement of the borrowing scolecophidians and the discovery of the four-legged *Tetrapodophis* as the earliest snake lineages (Martill et al., 2015, Reeder et al., 2015). The purpose of the present study is to investigate a well preserved fossil of a mosasaur replacement tooth for three characters thought to be crucial for the distinction between ophidian and varanoid dentitions (Zaher and Rieppel, 1999): (i) the recumbent or axial position of replacement teeth, (ii) the occurrence of resorption pits, and (iii) the presence of sockets or bony pedicels that anchor the tooth apex to the jaw. The direction of tooth eruption is one of the characters important for the varanoid/snake distinction of mosasaur dentitions. Replacement teeth in most squamates and crocodilians develop apical of the deciduous tooth, and teeth erupt in vertical direction and displace the erupted tooth, while snake replacement teeth are commonly found in an inclined or recumbent position. The mosasaur condition differs from the classic varanoid mode of tooth attachment because replacement teeth in some mosasaur fossils are preserved in an inclined position (Zaher and Rieppel, 1999, Rieppel and Kearney, 2005). These observations have prompted some authors to seek parallels between the inclined replacement teeth and pseudothecodont tooth implantation in mosasaurs with the recumbent replacement teeth and their synostotic mode of attachment in ophidians such as snakes as a morphological argument for the aquatic origin or snakes (Caldwell and Lee, 1997; Lee, 1997b).

The second character crucial to classify mosasaur dentitions among varanoids and snakes is the presence of resorption pits. In squamates, the resorption pit is a structure-less condensate that grows at the apex of a deciduous tooth through mineralized bone and dentin resorption to facilitate the growth of a new tooth within its boundaries. The terminology “resorption pit” is confusing as also the Howship's lacunae of cellular level osteoclast bone resorption are called resorption pits (Hefti et al., 2010). Replacement teeth in most squamates and in crocodilian archosaurs develop within resorption pits of deciduous teeth (Edmund, 1960; Martin et al., 1980; Zaher and Rieppel, 1999), while snake teeth develop in the free connective tissue between tooth bearing elements and oral mucosa without any bony encasement (Lee, 1997b; Rieppel, 1980; Vonk et al., 2008). From a functional perspective, the formation of resorption pits in squamates ensures removal of the deciduous tooth anchorage and also provides protection of the successional tooth within the already existing theca of the previous generation tooth.

The third feature important for the standing of mosasaurs in the varanoid/snake origin debate is the type of tooth implantation. Tooth implantation in most non-mosasauroid varanoids has been characterized as classical pleurodont while in snakes, tooth implantation is acrodont and teeth are ankylosed to the rim of the socket by means of a cementoid synostosis between the tooth apex and the tooth bearing element (TBE; Zaher and Rieppel, 1999). In contrast, tooth attachment in mosasaurs is lacking the non-mineralized periodontal ligament of mammalian and crocodilian teeth and instead features a mineralized ligamentous interface between cementum (bone of attachment) and interdental ridge (McIntosh et al., 2002; Luan et al., 2009b). Moreover, the interdental ridges between individual mosasaur teeth are relatively thinner and cover only a portion of the mosasaur root surface compared to the alveolar theca that surrounds most of the mammalian tooth root surface (Luan et al., 2009b). Thus, it makes sense to classify the mosasauroid mode of tooth implantation as pseudothecodont or subthecodont (Zaher and Rieppel, 1999).

The debate on Mosasaur-snake origins focuses on three features related to the dentition, the recumbent position of replacement teeth, the lack or presence of resorption pits, and the type of tooth implantation. To address key questions of tooth eruption and implantation in mosasaurs, we have analyzed a newly discovered and well-preserved specimen from the Texas shore of the Western Interior Seaway using ground sections as well as cone beam and microcomputer tomography. Our data provide novel insights into mosasaur tooth implantation and their phylogenetic implications.

## Materials and methods

### Samples used for comparative analysis

A jaw fragment of a late Cretaceous mosasaur featuring erupted and replacement teeth was obtained from an unspecified central Texas excavation site. Five representative extant species were chosen to provide a frame of reference for the position of the mosasaur replacement tooth: a rodent (mouse, *Mus musculus*), a marsupial (opossum, *Monodelphis domestica*), a crocodilian (American alligator, *Alligator mississippiensis*), a snake (ball python, *Python regius*), and a squamate (Iguana, *Iguana iguana*). Mouse, opossum, alligator, snake and iguana were sacrificed according to UIC animal care regulations and studies were approved by the UIC animal care committee. The Gila monster skull was from a private collection.

### X-ray tomography of calcified tissues

The position of replacement teeth vs. the position of already erupted teeth was assessed in all six extant species using micro-CTs (Scanco Model 40) at 70 kV for optimal imaging of teeth. Tomographic data of the jaw fragment were obtained using an iCAT cone beam tomograph (Imaging Sciences International LLC, Hatfield, PA) at 120 kV, and individual images were segmented using Azivo (FEI Visualization) to visualize the orientation of the mosasaur replacement tooth relative to the erupted tooth.

### Ultrathin ground sections of mosasaur jaw fragments

The fossil mandible and teeth were sectioned using an EXAKT diamond band saw. Sections were mounted and polished to 40 μm thickness using an EXAKT ultrathin grinding system to assess the histological context of the replacement tooth in our mosasaur jaw fragment.

### Skull preparation, whole mount stains, and paraffin sections

Mature skulls from mouse, opossum, alligator, snake, and iguana were photographed in lateral position. For whole mount and paraffin preparations, animals were sacrificed according to UIC animal regulations and either fixed in 70% ethanol (whole mount stains) or 10% formalin (paraffin sections). For whole mount stains, jaws were fixed and dehydrated with ethanol and acetone, stained with alcian blue and alizarin red S for 2 days, immersed in 0.5% potassium hydroxide solution, and stored in 80% glycerol. For paraffin sections, tissues were fixed in formalin, dehydrated using a graded series of ethanols, embedded in paraffin, and cut in 5 μm thick sections. Sections were stained using Mallory's trichrome stain.

## Results

### The sampled mosasaur replacement tooth was inclined at a 28° angle in distal direction, surrounded by an undistorted bony crypt, and located within a deciduous resorption pit

**Figure 1:**
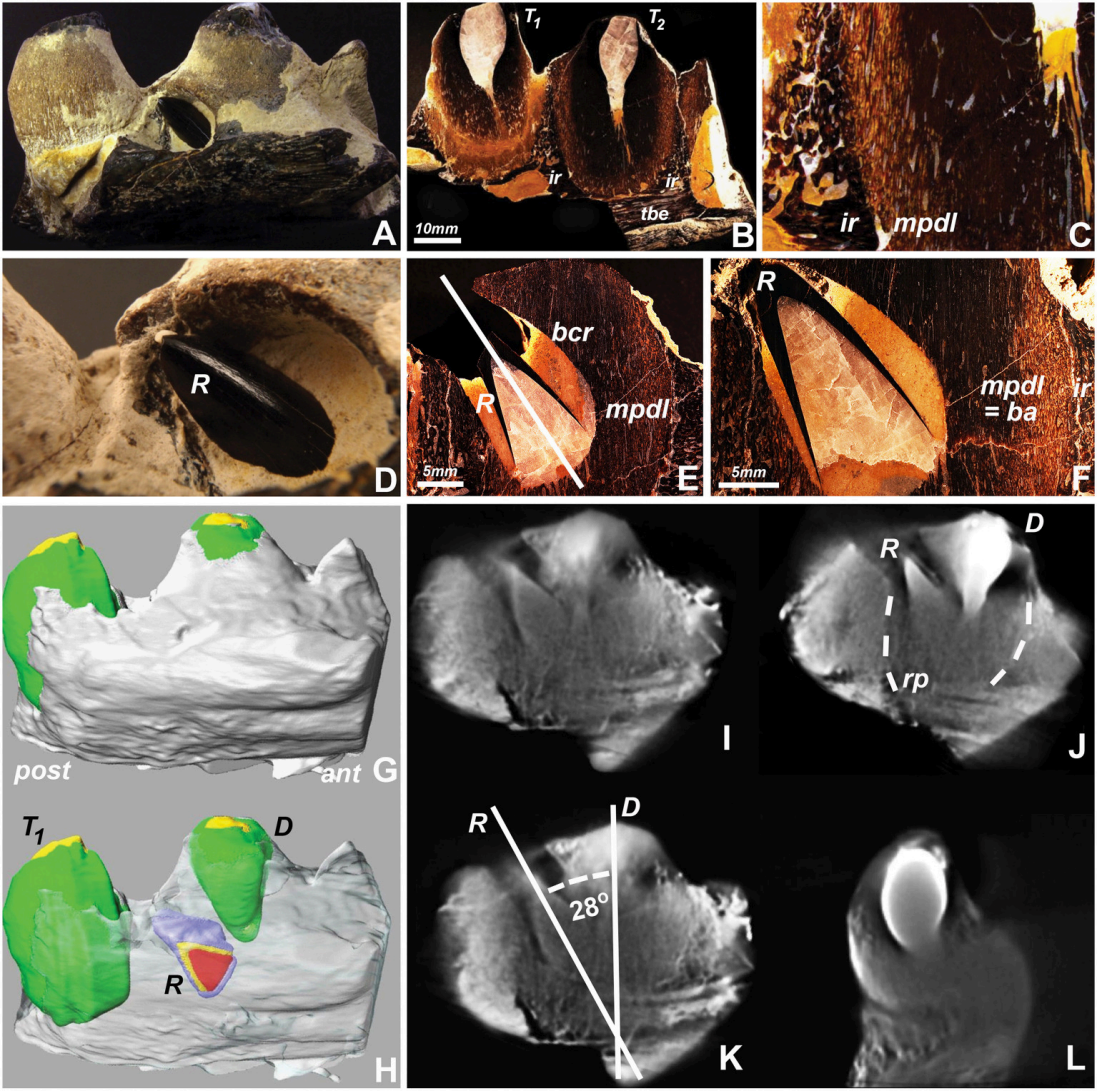
**Replacement tooth position in a fossil Mosasaur jaw fragment from central Texas**. **(A,D)** are macrographic images of the intact fragment, **(B,C,E,F)** are ultrathin ground sections, **(G,H)** are 3D-reconstructions of stacked tomographs highlighting individual tooth positions, and **(I–L)** are individual tomographs illustrating the relationship between the center tooth, its replacement tooth, and surrounding socket. **(H)** was generated by digitally removing the superficial set of tomographs and coloring the replacement tooth (R), and **(I–L)** are individual cone beam tomographs generated while rotating the individual jaw fragment during the 3D tomography rendering process. The interrupted lines in **(J)** indicate the boundaries between the tooth-pedicel complex and the pseudotheca formed by the adjacent interdental ridges. Abbreviations: T_1_ and T_2_, neighboring teeth; ir, interdental ridge; tbe, tooth bearing element; mpdl, mineralized periodontal ligament; pedicel, or ba, bone of attachment; R, replacement tooth; E, Deciduous tooth; bcr, bony crypt of the replacement tooth R; rp, resorption pit; ant, anterior (mesial); post, posterior (distal). The mpdl descriptor in **(E,F)** refers to the pedicel of the replacement tooth, while the mpdl in **(C)** represents the pedicel of the exfoliating tooth T_2_.

To conduct a detailed analysis of mosasaur tooth eruption and implantation, a well-preserved specimen from North Texas was analyzed using ground sections and micro-computed tomography. Macroscopic analysis identified the replacement tooth as a black-colored conical object protruding between the apex of the adjacent sand-colored erupted tooth and the dark brown-colored tooth-bearing element (Figures 1A,D). Ground sections prepared in a plane labial from the replacement tooth reveal an intact interdental ridge and tooth attachment apparatus (Figures 1B,C). The erupting tooth was surrounded by its own bony crypt and associated pedicel (a mineralized periodontium, Figures 1E,F). The inclined direction of the replacement tooth axis pointed toward the opening of the bony crypt (Figure 1E), indicating that the tooth had not rotated within the crypt. There was no tissue compression or distortion visible (Figures 1B,C,E,F), suggesting that the inclination of the replacement tooth was not a result of postmortem mechanical distortion or deformation.

Large scale cone beam tomographs of the 8 cm long mosasaur jaw fragment illustrated the position of the replacement tooth adjacent to the apex of the deciduous tooth (Figure 1H vs. Figure 1G). Individual cone beam tomographs were used to calculate the 28° distal inclination angle of the replacement tooth when measured against the vertical axis of the deciduous tooth (Figure 1K) and to visualize the spatial relationship between both teeth from various angles when rotated in horizontal direction (Figures 1I–L). The individual tomographs also illustrated the location of the replacement tooth within an electron lucent resorption pit. In this tomograph, both replacement tooth and deciduous tooth were positioned within the same pseudotheca formed by the electron dense interdental ridges (Figure 1J).

### On-axis and recumbent modes of tooth replacement in amniotes

**Figure 2:**
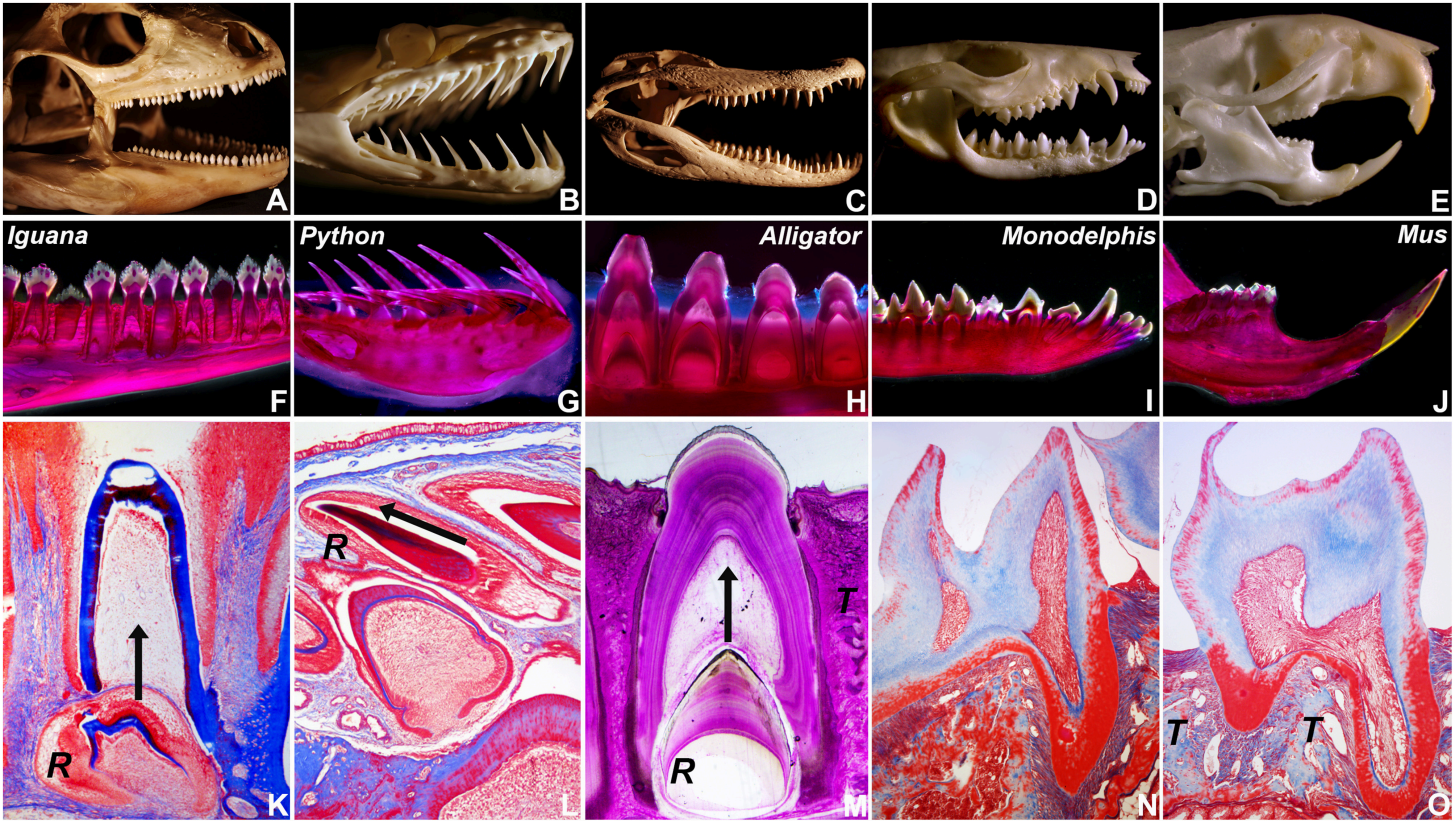
**Tooth shape and succession in select amniotes. (A–E)** are skull photographs, **(F–J)** are macrographs of whole mount stains, and **(K–O)** are micrographs of histological preparations based on the following specimen: **(A,F,K)** Green iguana (*Iguana iguana*), **(B,G,L)** Ball python (*Python regius*), **(C,H,M)** American alligator (*Alligator mississippiensis*), **(D,I,N)** Short-tailed opossum (*Monodelphis domestica*), and **(E,J,O)** house mouse (*Mus musculus*). (R) indicates the position of the replacement tooth **(K–M)**, (T) labels the alveolar bone theca in alligator and mouse **(M,O)**, and the arrows represent the direction of tooth eruption **(K–M)**.

To ask how mosasaur tooth replacement relates to tooth replacement mechanisms in various amniotes, tooth eruption and implantation were examined in five distinct amniote species on a macroscale and microscale level, including a squamate (Iguana, *I. iguana*), a snake (ball python, *P. regius*), a crocodilian (American alligator, *A. mississippiensis)*, a marsupial (opossum, *M. domestica*), and a rodent (mouse, *M. musculus*)(Figure 2). For macroscopic comparison, skulls of all five species were photographed (Figures 2A–E). Individual tooth rows including replacement teeth and their relationship to the jaw bone were visualized using whole mount stains (Figures 2F–J). Histological preparations of tooth replacement or individual teeth in mammalian dentitions were illustrated using Mallory stains (Figures 2K–O). The replacement dentition in the *Iguana* was positioned immediately apical of the deciduous erupted tooth, suggesting eruption of the replacement tooth in vertical direction (Figures 2F,K). Replacement teeth in the python were positioned at a ~15° inclination when compared to the horizontal jaw bone axis, while erupted teeth were inclined at an angle between ~35 and 60° in distal direction (Figure 2G). The histological section of a *P. regius* replacement dentition showed numerous replacement teeth prior to eruption and implantation (Figure 2L). None of these teeth demonstrated evidence of a mineralized pseudotheca surrounding the apical portion of the developing tooth (Figure 2L). In contrast, alligator (*A. mississippiensis*) tooth roots were surrounded by bony sockets (Figures 2H,M), and replacement teeth developed in tandem with partial resorption of the apical dentin/pulp complex of the deciduous tooth (Figure 2M). The two mammals employed in the present study were characterized by monophyodont dentitions, and there was no evidence of tooth replacement *per-se*. However, the frontal incisors of the *Monodelphis* were inclined at a 30–60° angle (Figure 2I), and the continuously erupting mouse incisor was positioned at a 60° angle (Figure 2J) illustrating that modes of tooth replacement and individual tooth inclination vary greatly among vertebrate species.

The Gila monster *Heloderma suspectum* employs a combination of drift and rotation to position the replacement tooth in alignment with the deciduous tooth.

**Figure 3:**
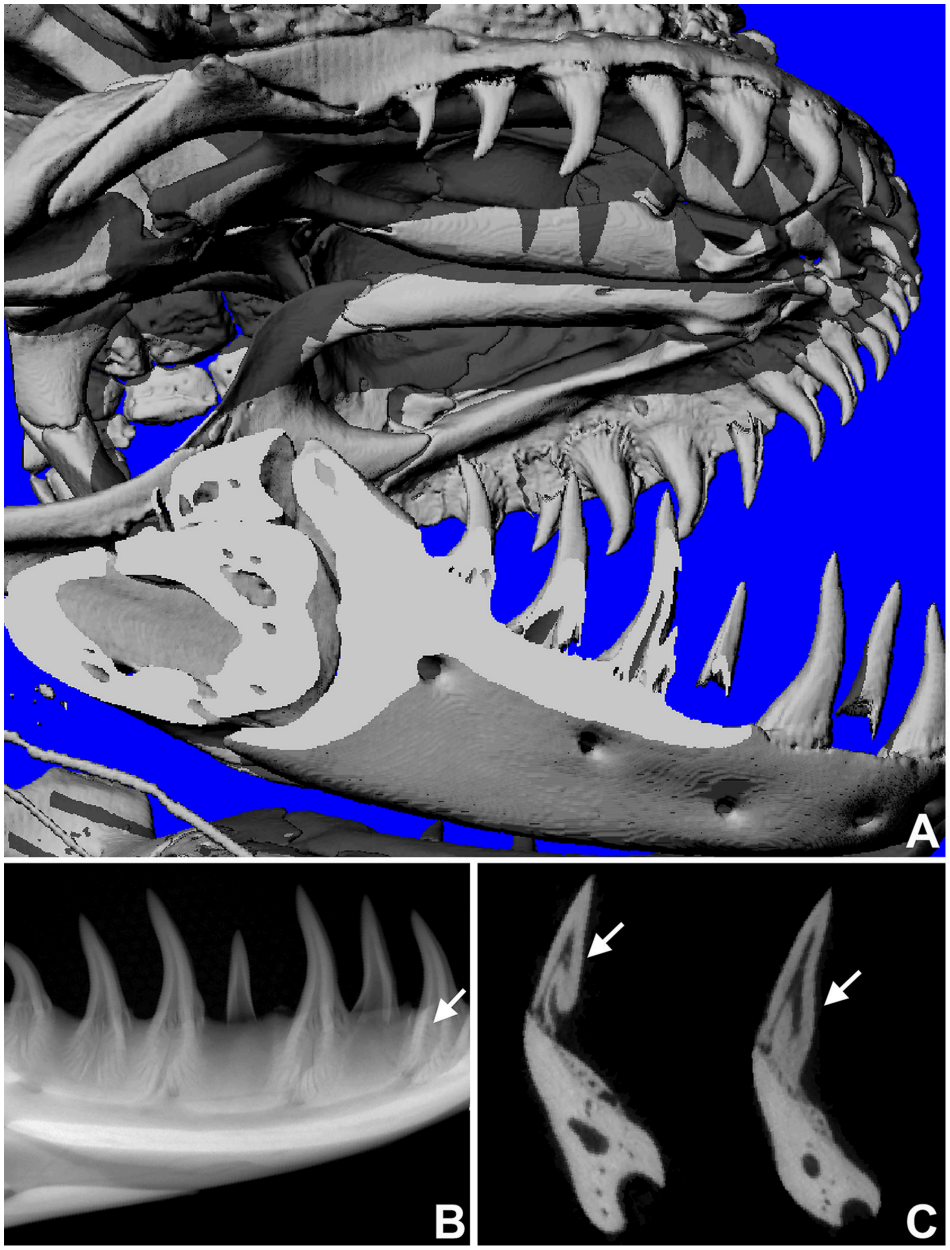
**Replacement tooth position in the Gila monster (***Heloderma suspectum***). (A)** is a microCT image of the entire skull, **(B,C)** are radiographs in sagittal **(B)** and transverse **(C)** orientation, revealing the position of the replacement tooth (arrows).

One of the key arguments for mosasaur-snake affinities is the inclined orientation frequently observed in mosasaur replacement teeth, which at least in terms of its angular position resembles the recumbent position of replacement teeth in snakes. In contrast, most squamates other than snakes feature on-axis vertical tooth replacement modes. To ask the question whether off-axis tooth replacement also occurs in other varanoids, the skull of a Gila monster, *H. suspectum*, was subjected to 3D microtomography and X-ray analysis (Figure 3). X-ray images and micro-CTs revealed a replacement tooth in the lower jaw located slightly distal (posterior) of the deciduous tooth (Figures 3A–C). As demonstrated by X-ray analysis, radiolucent areas of the newly formed tooth overlapped with those of the deciduous tooth, suggesting that the replacement tooth formed inside of a resorption pit within the deciduous tooth (Figure 3C).

### The relationship between vertical tooth axis orientation and replacement tooth position varies among amniotes

**Figure 4:**
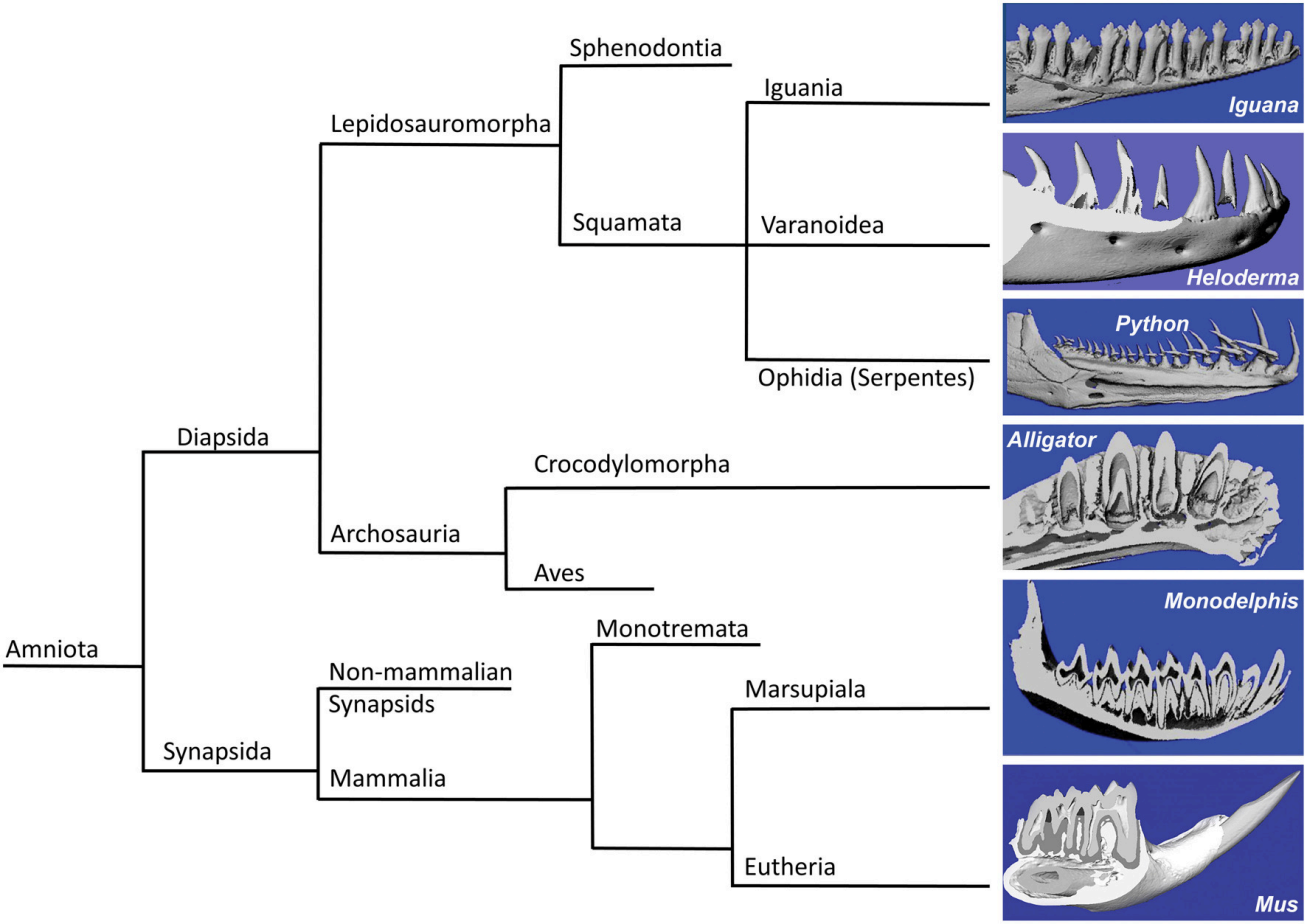
**Consensus phylogeny illustrating the relationships of the species included in the present analysis**. The left side of the diagram illustrates the phylogenetic relationship between the species studies in the present analysis, including green iguana (*Iguana iguana*), ball python (*Python regius*), Gila monster (*Heloderma suspectum*), American alligator (*Alligator mississippiensis*), short-tailed opossum (*Monodelphis domestica*), and house mouse (*Mus musculus*). Three-dimensional micro-CT reconstructions of the tooth rows are presented on the right side of the illustration.

In the present study we asked the question whether mosasaur tooth replacement and successional tooth implantation mechanisms are identifying characters that establish mosasaurs as a family unequivocally positioned within squamates and define its relationship with other squamates such as snakes and varanoids (Figure 4). For this purpose, fossil mosasaur jaw fragment preparations were compared against skeletal and histological preparations of extant amniotes, representing various groups, including varanoids, snakes, crocodilians, marsupials, and rodents. Vertebrate systematics place Mosasaurs together with other varanoids (e.g., the iguana and the Gila monster used in the present study) and snakes (e.g., the python used in the present study) within the order of squamates (Squamata), which in turn belongs to the superorder lepidosaurs. Together with the archosaurs (represented here by the alligator), lepidosaurs are part of the diapsid clade, while the two mammals studied here, the Monodelphis (a marsupial) and the mouse (a rodent), belong to the synapsid clade. Both diapsids and synapsids are amniotes.

Three-dimensional micro-CT reconstructions of tooth bearing jaw fragments of all six species illustrate the orientation of tooth axis and replacement teeth in relationship to the jaw bone (Figure 4, right panel). These reconstructions illustrate that the replacement teeth in iguana and alligator were oriented in vertical direction, while the python replacement teeth were positioned at a 60° distal inclination to the erupted teeth. In another varanoid studied here, the Gila monster *Heloderma*, tooth crowns were slightly (~10–20°) inclined in distal direction when compared to the horizontal jaw axis, and replacement teeth were located posterior (distal) of the fully erupted tooth (Figure 4). In contrast to the four diapsids studied here, the dentition of the two mammals investigated (*Monodelphis* and *Mus*) lacked classic replacement teeth (monophyodont dentition), and only the mouse incisor featured permanent eruption and was inclined at a 60° angle when compared to the erupted molars (Figure 4).

The rotational drift employed during mosasaur tooth eruption and implantation resembles tooth eruption/implantation mechanisms found in Heloderma, while other varanoids such as the Iguana feature on-axis tooth eruption/replacement, and snakes such as the Python develop recumbent replacement teeth.

**Figure 5:**
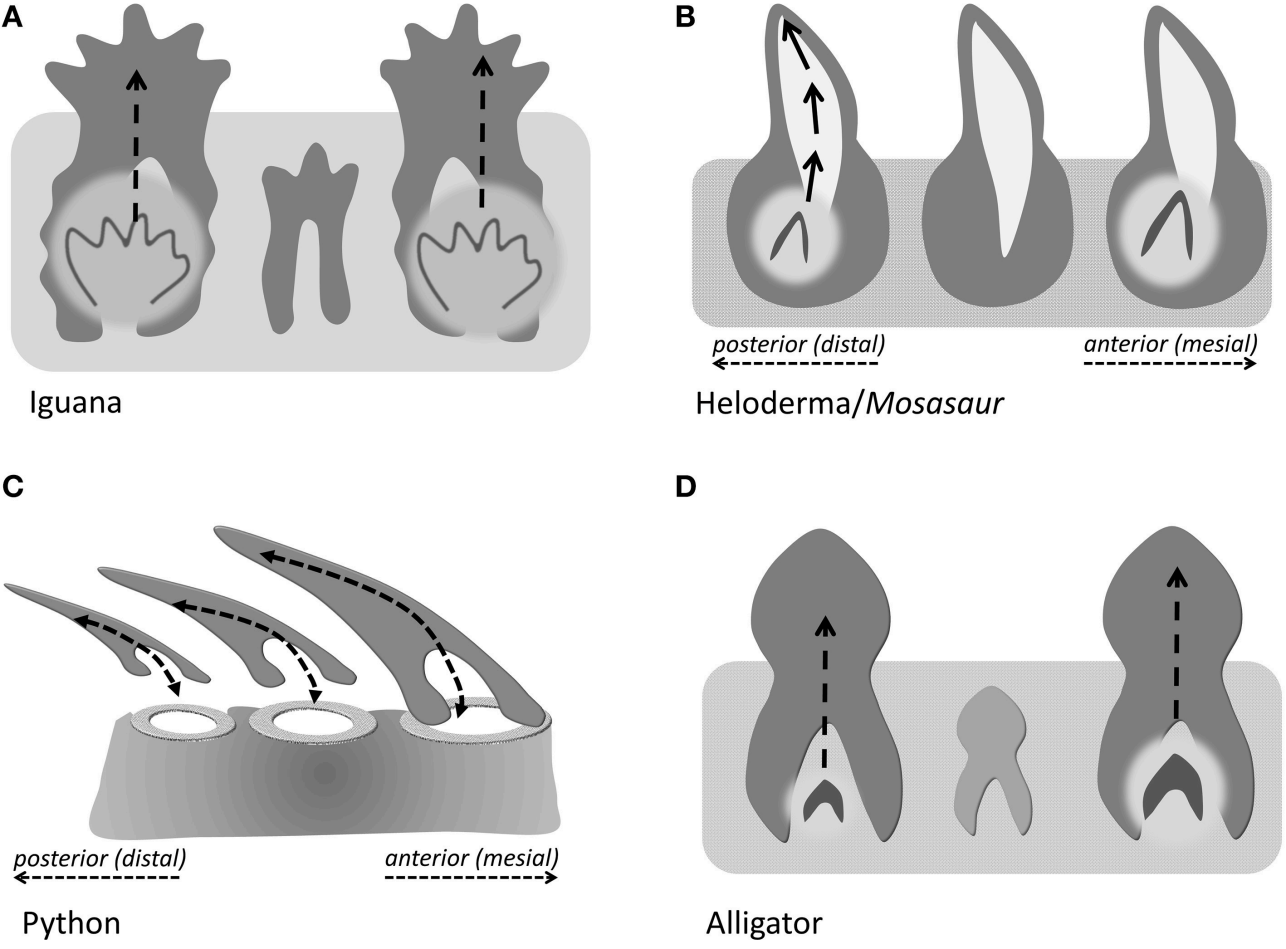
**Direction of tooth eruption in select amniotes**. Sketch illustrating the common straight upright eruption pattern in iguana and alligator **(A,D)**, the recumbent position of replacement teeth in snakes **(C)**, and the rotational shift that the replacement tooth undergoes when erupting to replace its predecessor tooth **(B)**, as a proposed mechanism in Helodermitidae and Mosasaurs.

In the present study, we distinguish between three different modes of tooth eruption and implantation in diapsid amniotes (Figure 5). Squamates and crocodilia favor on axis tooth replacement with the replacement tooth germ developing within a resorption pit at the apical portion of the deciduous tooth (Figures 5A,D). In most squamates, replacement teeth are then implanted in a pleurodont fashion, while crocodilian archosaur teeth are implanted into a *theca*. We propose that the replacement teeth in evolved varanoids such as *Mosasauridae* and *Helodermitidae* develop within resorption pits and then move into the position of the deciduous teeth through distal drift and a rotational movement (Figure 5B). Finally, ophidian tooth development differs from mosasaurian and varanoid tooth development as snake teeth develop within the supramandibular odontogenic connective tissue and only after completion of crown formation attach to the tooth bearing element through synostosis (ankylosis; Figure 5C).

## Discussion

In the present study a fossil mosasaur jaw fragment containing an erupting tooth was analyzed using ultrathin ground sections and cone beam 3D computer tomography to compare mosasaur tooth eruption and implantation with tooth replacement mechanisms in extant amniotes, including an iguana, a python, a Gila monster, an alligator, a *Monodelphis* marsupial, and a mouse. In the fossil Mosasaur jaw fragment studied here, the replacement tooth was inclined, and there was no evidence of postmortem displacement as the surrounding bone microstructure was intact. Cone beam-CT analysis revealed the presence of a resorption pit and indicated that both the deciduous tooth and its replacement tooth were positioned within the same pseudo-theca. Ultrathin ground sections confirmed the presence of a pedicel formed by a mineralized periodontal ligament, connecting the mosasaur tooth root with the adjacent interdental ridge to provide a pseudo-thecodont attachment. The combination of drift and rotation as the proposed mode of Mosasaur tooth replacement resembled similar tooth replacement/implantation movements in the extant Gila monster *H. suspectum*. In contrast, other amniotes studied here featured vertical on-axis tooth replacement, while recumbent replacement teeth were only detected in our python sample, and mammals exhibited a variety of tooth axis orientations.

### Replacement tooth rotation: Artifact, ophidian, or varanoid character?

The mosasaur replacement tooth examined in the present study was observed in an inclined position similar to replacement teeth previously described in a number of mosasaurs including *Leiodon, Platecarpus, Tylosaurus*, and *Clidastes* (Lee, 1997a). Moreover, our ultrathin ground sections and 3D cone beam tomographs demonstrated that the position and shape of the bony crypt opening matched the inclination angle of the replacement tooth. These ground sections and tomographs did not provide any evidence of postmortem displacement or deformation, but rather demonstrated that inclined replacement teeth and their integument were fairly well-preserved. Thus, our evidence suggests that the inclined position of our mosasaur replacement tooth is representative of the actual position of these teeth in at least some Cretaceous mosasaur species.

However, not only snakes and mosasaurs have off-axis and lingual replacement teeth that are often inclined or recumbent, but also closely related extant varanoids such as the Gila monster *Heloderma* are characterized by similar rotational and off-axis replacement teeth as demonstrated in the present study and as previously described by Smith (1958, note Figure 6). Moreover, inclined or recumbent tooth positions are prominent in a broad range of amniotes including some mammals (rodents, marsupials). Thus, a recumbent tooth position is not necessarily a defining character for synaptomorphic mosasaur-snake affinities, but rather a homoplastic character that occurs in a number of amniotes. From a morphological position alone, which is the basis of Lee's argument (Lee, 2005), Mosasaur-Heloderma tooth eruption/implantation synaptomorphies are more plausible than those between mosasaurs and snakes, suggesting that mosasaurs and Helodermatidae may safely be grouped among the varanoids, as suggested by Zaher and Rieppel (1999). A possible relationship between mosasaurs and Helodermatidae is also supported by earlier studies on tongue morphologies (Schulp et al., 2005) and by the posterolingual (distal) replacement tooth position described in the present study in both the *Heloderma* specimen and the mosasaur fragment. One benefit of an inclined or somewhat recumbent position would be a longer period of protected development for the replacement tooth, which might provide an important advantage for mosasaurs when it comes to the preservation of a fairly complete tooth row as a prerequisite for preying on large organisms with calcareous shells.

### The resorption pit: Unprotected *de novo* tooth formation or protected replacement of existing teeth?

Our study indicated that the developing mosasaur replacement tooth was surrounded by a resorption pit within the boundaries of the deciduous tooth. Replacement tooth and deciduous tooth were located within the same pseudotheca formed by the interdental ridges between adjacent teeth. As demonstrated in the present study, mosasaurian, iguanid, and crocodilian replacement teeth developed within resorption pits generated at the apex of the previous generation tooth and at the expense of the deciduous tooth mineral substance, while the python snake teeth studied here do not. Specifically, developing python tooth germs were not surrounded by any mineralized tissue as demonstrated in the present study, making it impossible for resorption pits to form. In terms of the mosasaur-sake debate, Lee was aware that mosasaurs and many other squamates form resorption pits for tooth replacement, while there are no resorption pits in snake teeth and replacement teeth lie outside the jawbone throughout their entire development (Lee, 1997a). It is thus surprising that he gave mosasaur-snake synaptomorphies serious consideration. The presence of resorption pits in mosasaurs and varanoids vs. their absence in snakes as documented in the present study certainly argues in favor of mosasaur-varanoid homologies.

### Tooth implantation: Pseudotheca or synostosis?

In terms of tooth implantation, our specimen demonstrated anchorage of the bulbous mosasaur root cementum by means of a mineralized periodontal ligament in a bowl-shaped groove between interdental ridges as described earlier (Luan et al., 2009b). This mode of attachment resembled thecoid forms of tooth attachment but fell short of true thecodont attachment because of the mineralized state of the ligament and because of the shallow interdental ridges that only provided incomplete anchorage for the protruding tooth. As such, we have chosen the term pseudothecodont to define the mode of mosasauroid tooth attachment as an intermediary between ankylosis and ligamentous thecodont attachment. Our term pseudothecodont is based on the ligamentous structure and mineralized composition of the mosasaur attachment tissue that interfaces between interdental ridges and the bulbous root cementum (Luan et al., 2009b), and describes similar forms of implantation as identified by the earlier terms “subthecodont” or “ankylosed thecodont” (Zaher and Rieppel, 1999). Thus, our data indicate that mosasaur teeth are truly unique and can neither be compared with the cementoid ankylosis to the socket rim as it occurs in snakes nor with the pleurodont ankylosis that is typical for most extant varanoids.

### The origin of snakes—a flashback from the bone wars?

In the present study we have demonstrated (i) that the presence of inclined replacement teeth is not unique to mosasauroid varanoids but also occurs in helodermatoid squamates and is therefore is not a synaptomorphic character exclusive for mosasaurs and snakes, (ii) that the occurrence of resorption pits as structural basis for replacement tooth formation within the deciduous precursor theca is a character found both in mosasaurs and varanoids, but not in snakes, supporting mosasaur-varanoid affinities, and (iii) that the pseudothecodont mode of tooth implantation in mosasaurs is different from the pleurodont type of tooth attachment as it occurs in many varanoids and from the ankylotic synostosis on the rim of the TBE as it is representative of most snakes, suggesting that mosasaurian pseudothecodont tooth implantation is an autopomorphic character for mosasaurs alone. Together, our study suggests that the mosasaur tooth apparatus is distinct from varanoids and snakes and shares common features with both groups, while the pseudothecodont implantation of mosasaurs does not occur in either of the other two groups.

The original concept of mosasaur-snake affinities goes back to Edward D. Cope (1840–1897), who together with Othniel Charles Marsh (1831–1899) was known for his fossil-finding expeditions during the Bone Wars. Cope argued for a systematic relationship between mosasaurs and snakes based on common traits in their lower jaws, including a free mandibular symphysis and a straight vertical splenial-angular joint, allowing for a large gape to facilitate feeding on large prey (Cope, 1869, 1872; Lee, 1997b; Luan et al., 2009b). Cope's account was originally disputed by Owen (1877, 1878), but later adopted by Lee (1997a,b) based on two derived traits, (i) the presence of recumbent replacement teeth and (ii) the establishment of a discrete socket under each tooth (Lee, 1997a). For Cope, the transformation of marine mosasaurs into modern snakes was just one example for his Neo-Lamarckian school of thought, according to which organisms slowly evolve over time and pass on their fittest traits toward their offspring (Cope, 1889).

Proponents of the snake-mosasaur common ancestor school of thought have invoked the discovery of fossil marine snakes with limbs as supportive of mosasaur-snake origins (Caldwell and Lee, 1997). However, a close relationship between snakes and monitor lizards has been refuted by recent DNA evidence (Vidal and Hedges, 2004), and an extensive study including 161 squamate species and up to 44 nuclear genes reported a paraphyletic relationship between scolecophidians and other snakes, supporting the burrowing hypothesis of snake origins and at the same time rejecting the hypothesis of marine origins of snakes (Wiens et al., 2012).

It appears as if the mosasaur feeding apparatus was uniquely specialized to anchor large-sized teeth within powerful jaws (TBEs) and to provide optimum protection for replacement teeth within a bony crypt that develops within a resorption pit at the apex of its predecessor. While the recumbent orientation of snake dentitions facilitates retention of prey and prevents a possible escape of captured organisms, mosasaur dentitions appear to be adept at feeding on a broad range of food, including a diet of large fish, ammonites, sea turtles, and crustaceans (Polcyn et al., 2014). Crushing the bones and mineralized shells of such prey would be traumatic for developing teeth, unless they were protected by bony crypts or the covers of already erupted teeth with a rigid yet flexible attachment apparatus (Luan et al., 2009b). It seems as if Cope was captivated by the morphological similarities between large angioform animals such as mosasaurs and snakes, and the skeletal similarities that supported his Neo-Lamarckian views, while he appeared to have overlooked the selective pressures in late Cretaceous oceans.

## Author contributions

ML, GC, XL, KG, CG prepared samples and conducted experiments; XL analyzed data and generated images; DR, RM, XL, and TD analyzed data and wrote the manuscript.

### Conflict of Interest Statement

The authors declare that the research was conducted in the absence of any commercial or financial relationships that could be construed as a potential conflict of interest.
